# Supplementary material for: Deletion of wfs1 Impairs Oligodendrocyte Precursor Cells Dorsal Distribution and Myelination Through the wfs1-hmgcs1 Axis in Zebrafish
Source: Biology (Basel). 2026 Aug 16;15(16):1402. doi: 10.3390/biology15161402 (PMC13510151; doi:10.3390/biology15161402)
Supplement: Supplementary file 1 [file biology-15-01402-s001.zip › File S1-Transcriptome sequencing data and differentially expressed genes (DEGs) in wfs1-- zebrafish at 5 dpf/2.KEGG/wfs1KOvsWT/GSEA/WT/LYSINE_DEGRADATION/LYSINE_DEGRADATION(DRE00310).html]

Details for gene set LYSINE\_DEGRADATION(DRE00310)[GSEA]

|  || Dataset | deg.sample |
| Phenotype | sample.cls |
| Upregulated in class | WT |
| GeneSet | LYSINE\_DEGRADATION(DRE00310) |
| Enrichment Score (ES) | -0.23567727 |
| Normalized Enrichment Score (NES) | -1.2279646 |
| Nominal p-value | 0.10934394 |
| FDR q-value | 0.51877177 |
| FWER p-Value | 1.0 |
Table: GSEA Results Summary

  

Fig 1: Enrichment plot: LYSINE\_DEGRADATION(DRE00310)      
 Profile of the Running ES Score & Positions of GeneSet Members on the Rank Ordered List

  

| PROBE | DESCRIPTION (from dataset) | GENE SYMBOL | GENE\_TITLE | RANK IN GENE LIST | RANK METRIC SCORE | RUNNING ES | CORE ENRICHMENT || 1 | 100003615 | prdm2a |  |  | 778 | 2.426 | 0.0183 | No |
| 2 | 556077\_1 | plod3 |  |  | 1001 | 2.091 | 0.0481 | No |
| 3 | 393860 | gcdha |  |  | 1119 | 1.976 | 0.0792 | No |
| 4 | 368239 | aldh2.2 |  |  | 1221 | 1.881 | 0.1092 | No |
| 5 | 553401 | hadhaa |  |  | 1608 | 1.593 | 0.1251 | No |
| 6 | 447920 | aldh3a2b |  |  | 1830 | 1.444 | 0.1436 | No |
| 7 | 402830 | ehmt1b |  |  | 2669 | 1.121 | 0.1370 | No |
| 8 | 556535 | setd1a |  |  | 2902 | 1.058 | 0.1483 | No |
| 9 | 368262\_1 | dlst |  |  | 2921 | 1.056 | 0.1663 | No |
| 10 | 572649 | bbox1 |  |  | 2922 | 1.056 | 0.1849 | No |
| 11 | 326906 | suv39h1b |  |  | 4156 | 0.801 | 0.1603 | No |
| 12 | 30643 | acat2 |  |  | 4174 | 0.798 | 0.1738 | No |
| 13 | 571274 | setd1bb |  |  | 4438 | 0.755 | 0.1788 | No |
| 14 | 796720 | si:ch211-263k4.2 |  |  | 4504 | 0.744 | 0.1899 | No |
| 15 | 100000859 | ehhadh |  |  | 4565 | 0.737 | 0.2010 | No |
| 16 | 564592 | nsd1b |  |  | 4750 | 0.712 | 0.2077 | No |
| 17 | 100005587 | aldh9a1a.1 |  |  | 5134 | 0.663 | 0.2073 | No |
| 18 | 497407 | mecom |  |  | 5585 | 0.628 | 0.2042 | No |
| 19 | 399481 | aldh9a1b |  |  | 6718 | 0.518 | 0.1778 | No |
| 20 | 541423 | smyd2a |  |  | 6906 | 0.500 | 0.1807 | No |
| 21 | 777635 | plod1a |  |  | 7064 | 0.486 | 0.1843 | No |
| 22 | 393462 | aldh2.1 |  |  | 8615 | 0.394 | 0.1425 | No |
| 23 | 572649\_1 | bbox1 |  |  | 8772 | 0.383 | 0.1443 | No |
| 24 | 100000026 | aldh3a1 |  |  | 8825 | 0.379 | 0.1494 | No |
| 25 | 558601 | pipox |  |  | 8826 | 0.379 | 0.1560 | No |
| 26 | 100004770 | setd8b |  |  | 8862 | 0.377 | 0.1616 | No |
| 27 | 572849 | suv420h1 |  |  | 9019 | 0.366 | 0.1631 | No |
| 28 | 101886516 | prdm6 |  |  | 9385 | 0.342 | 0.1577 | No |
| 29 | 796749 | si:ch73-54n14.2 |  |  | 9859 | 0.312 | 0.1483 | No |
| 30 | 793834 | hadhab |  |  | 10077 | 0.298 | 0.1467 | No |
| 31 | 568162 | kmt2cb |  |  | 12302 | 0.174 | 0.0798 | No |
| 32 | 568616 | smyd2b |  |  | 12307 | 0.173 | 0.0827 | No |
| 33 | 556229 | aass |  |  | 13019 | 0.139 | 0.0628 | No |
| 34 | 100004770\_2 | setd8b |  |  | 13531 | 0.114 | 0.0488 | No |
| 35 | 100004770\_1 | setd8b |  |  | 13643 | 0.109 | 0.0472 | No |
| 36 | 100000709\_1 | whsc1 |  |  | 13661 | 0.108 | 0.0486 | No |
| 37 | 567970 | setd1ba |  |  | 14027 | 0.091 | 0.0387 | No |
| 38 | 492710 | aldh16a1 |  |  | 14167 | 0.084 | 0.0358 | No |
| 39 | 566825 | kmt2bb |  |  | 14417 | 0.073 | 0.0293 | No |
| 40 | 751629 | setd8a |  |  | 14607 | 0.065 | 0.0245 | No |
| 41 | 100000709 | whsc1 |  |  | 14686 | 0.061 | 0.0231 | No |
| 42 | 560856 | kmt2ba |  |  | 14703 | 0.060 | 0.0236 | No |
| 43 | 335153 | setdb2 |  |  | 14765 | 0.057 | 0.0227 | No |
| 44 | 560793 | kmt2ca |  |  | 15000 | 0.046 | 0.0162 | No |
| 45 | 565862 | colgalt1 |  |  | 15218 | 0.036 | 0.0100 | No |
| 46 | 100536523 | kmt2d |  |  | 15707 | 0.011 | -0.0052 | No |
| 47 | 559560 | hykk.2 |  |  | 16258 | -0.009 | -0.0223 | No |
| 48 | 100036767 | plod2 |  |  | 16643 | -0.021 | -0.0340 | No |
| 49 | 100003546 | colgalt2 |  |  | 16683 | -0.022 | -0.0348 | No |
| 50 | 797296 | prdm2b |  |  | 16933 | -0.032 | -0.0421 | No |
| 51 | 563799 | ash1l |  |  | 17129 | -0.037 | -0.0476 | No |
| 52 | 569027 | smyd1b |  |  | 17254 | -0.043 | -0.0507 | No |
| 53 | 572849\_1 | suv420h1 |  |  | 17564 | -0.057 | -0.0595 | No |
| 54 | 321245 | smyd1a |  |  | 17615 | -0.059 | -0.0600 | No |
| 55 | 768131 | setdb1b |  |  | 17628 | -0.059 | -0.0593 | No |
| 56 | 569507 | smyd3 |  |  | 17678 | -0.061 | -0.0598 | No |
| 57 | 557048 | kmt2a |  |  | 17682 | -0.062 | -0.0588 | No |
| 58 | 569041\_1 | suv420h2 |  |  | 17759 | -0.065 | -0.0600 | No |
| 59 | 553292 | setdb1a |  |  | 17866 | -0.070 | -0.0621 | No |
| 60 | 100006350 | whsc1l1 |  |  | 18064 | -0.079 | -0.0669 | No |
| 61 | 445121 | hadh |  |  | 18086 | -0.080 | -0.0662 | No |
| 62 | 557979 | aadat |  |  | 18128 | -0.082 | -0.0660 | No |
| 63 | 368912 | echs1 |  |  | 18310 | -0.089 | -0.0701 | No |
| 64 | 794199 | setd2 |  |  | 18374 | -0.092 | -0.0705 | No |
| 65 | 100005761 | ehmt1a |  |  | 18425 | -0.095 | -0.0704 | No |
| 66 | 325395 | kmt2e |  |  | 18629 | -0.105 | -0.0749 | No |
| 67 | 768133 | ezh2 |  |  | 19275 | -0.134 | -0.0929 | No |
| 68 | 494076\_1 | dhtkd1 |  |  | 20185 | -0.174 | -0.1184 | No |
| 69 | 571274\_1 | setd1bb |  |  | 20890 | -0.209 | -0.1369 | No |
| 70 | 556077 | plod3 |  |  | 20985 | -0.215 | -0.1360 | No |
| 71 | 334197 | aldh7a1 |  |  | 21094 | -0.221 | -0.1355 | No |
| 72 | 567859 | si:ch211-114l13.7 |  |  | 21180 | -0.225 | -0.1343 | No |
| 73 | 569250 | ehmt2 |  |  | 21254 | -0.228 | -0.1325 | No |
| 74 | 445290 | acat1 |  |  | 21467 | -0.239 | -0.1350 | No |
| 75 | 559945 | dot1l |  |  | 21571 | -0.244 | -0.1339 | No |
| 76 | 100537860 | LOC100537860 |  |  | 21649 | -0.248 | -0.1320 | No |
| 77 | 664754 | ezh1 |  |  | 21872 | -0.260 | -0.1344 | No |
| 78 | 564575 | setmar |  |  | 21969 | -0.266 | -0.1327 | No |
| 79 | 393876 | prdm9 |  |  | 22175 | -0.277 | -0.1343 | No |
| 80 | 445198 | suv39h1a |  |  | 22342 | -0.286 | -0.1345 | No |
| 81 | 567970\_1 | setd1ba |  |  | 22766 | -0.311 | -0.1423 | No |
| 82 | 100000709\_2 | whsc1 |  |  | 22849 | -0.316 | -0.1393 | No |
| 83 | 100006238 | aldh9a1a.2 |  |  | 23161 | -0.338 | -0.1431 | No |
| 84 | 556086 | nsd1a |  |  | 24921 | -0.442 | -0.1907 | No |
| 85 | 399479 | dldh |  |  | 25027 | -0.448 | -0.1861 | No |
| 86 | 559635 | hykk.1 |  |  | 25689 | -0.501 | -0.1980 | No |
| 87 | 436729 | setd7 |  |  | 26299 | -0.557 | -0.2074 | No |
| 88 | 557205 | phykpl |  |  | 26437 | -0.571 | -0.2016 | No |
| 89 | 796749\_1 | si:ch73-54n14.2 |  |  | 26567 | -0.585 | -0.1954 | No |
| 90 | 555271 | tmlhe |  |  | 27496 | -0.672 | -0.2128 | No |
| 91 | 323653 | aldh3a2a |  |  | 28024 | -0.745 | -0.2162 | No |
| 92 | 494076 | dhtkd1 |  |  | 28644 | -0.846 | -0.2208 | Yes |
| 93 | 558337\_1 | cercam |  |  | 28814 | -0.881 | -0.2106 | Yes |
| 94 | 450003 | gcdhb |  |  | 29282 | -0.995 | -0.2077 | Yes |
| 95 | 100006238\_1 | aldh9a1a.2 |  |  | 29971 | -1.231 | -0.2077 | Yes |
| 96 | 402830\_1 | ehmt1b |  |  | 30364 | -1.423 | -0.1950 | Yes |
| 97 | 393860\_1 | gcdha |  |  | 30857 | -1.817 | -0.1784 | Yes |
| 98 | 368262 | dlst |  |  | 30863 | -1.822 | -0.1465 | Yes |
| 99 | 556535\_1 | setd1a |  |  | 30934 | -1.895 | -0.1153 | Yes |
| 100 | 100005587\_1 | aldh9a1a.1 |  |  | 31308 | -2.447 | -0.0840 | Yes |
| 101 | 100536795 | wu:fb14d09 |  |  | 31372 | -2.574 | -0.0406 | Yes |
| 102 | 100003615\_1 | prdm2a |  |  | 31589 | -3.227 | 0.0095 | Yes |
Table: GSEA details [plain text format]

  

Fig 2: LYSINE\_DEGRADATION(DRE00310)      
 Blue-Pink O' Gram in the Space of the Analyzed GeneSet

  

Fig 3: LYSINE\_DEGRADATION(DRE00310): Random ES distribution      
 Gene set null distribution of ES for **LYSINE\_DEGRADATION(DRE00310)**
